# Macromolecular refinement by model morphing using non-atomic parameterizations

**DOI:** 10.1107/S205979831701350X

**Published:** 2018-02-01

**Authors:** Kevin Cowtan, Jon Agirre

**Affiliations:** a Department of Chemistry, University of York, York, England

**Keywords:** refinement, low resolution, computational methods

## Abstract

Refinement is a critical step in the determination of a model which explains the crystallographic observations and thus best accounts for the missing phase components. The scattering density is usually described in terms of atomic parameters; however, in macromolecular crystallography the resolution of the data is generally insufficient to determine the values of these parameters for individual atoms. Stereochemical and geometric restraints are used to provide additional information, but produce interrelationships between parameters which slow convergence, resulting in longer refinement times. An alternative approach is proposed in which parameters are not attached to atoms, but to regions of the electron-density map. These parameters can move the density or change the local temperature factor to better explain the structure factors. Varying the size of the region which determines the parameters at a particular position in the map allows the method to be applied at different resolutions without the use of restraints. Potential applications include initial refinement of molecular-replacement models with domain motions, and potentially the use of electron density from other sources such as electron cryo-microscopy (cryo-EM) as the refinement model.

## Abstract 2

A method is described for the refinement of an electron-density model against a set of structure-factor observations which does not rely on atomic parameters. The effective level of detail in the parameterization can be varied to ensure that the refinement is well determined at any resolution supported by the data.

## 1. Introduction   

The primary aim of most X-ray crystallographic experiments is to obtain a model of the scattering matter of the crystal in terms of atomic coordinates, from which the bonding and therefore the chemistry may be deduced. For macromolecules, differences between crystal cells and thermal motion within a crystal cell generally limit the resolution of the diffraction pattern so that atomic centres can not be accurately distinguished. In practice, an approximate model must be constructed by manual or automatic examination of the electron density, and then the parameters of the atomic model – coordinates, isotropic or anisotropic displacement parameters and occupancy – must be refined to best explain the observed diffraction amplitudes or intensities, yielding plausible estimates for the phase components that are lost in the experiment.

The refinement is performed in such a way to minimize some target function, which may be a least-squares difference between the observed structure-factor amplitudes and values calculated using the atomic model parameters (Driessen *et al.*, 1989 ▸; Sheldrick, 2007 ▸) or, in more recent years, a negative log-likelihood function (Murshudov *et al.*, 1997 ▸; Blanc *et al.*, 2004 ▸; Afonine *et al.*, 2012 ▸). The minimization may be performed directly in reciprocal space, by adjusting the model parameters to reduce the target function. Alternatively, the derivative of the target function with respect to the model structure factors can be determined in order to identify the direction in which changes to the model should alter the model structure factors. The Fourier transform of these gradients gives rise to a form of difference map, weighted according to the type of target function being used (Henderson & Moffat, 1971 ▸; Read, 1986 ▸). The refinement calculation may therefore be considered a problem in adjusting the model parameters to minimize the features of the difference map.

In macromolecular crystallography, the limited resolution of the data mean that the observation-to-parameter ratio may not be significantly greater than 1, and therefore additional geometric restraints are used between atoms to ensure that the problem is well determined and that the results are not overly influenced by the noise in the data. The X-ray observations have a similar influence on every model parameter (at least in the case of atomic coordinates), since every atom contributes to every structure factor. However, the geometrical restraints, and in particularly bond lengths which are tightly restrained, introduce strong correlations between different parameters. As a result the refinement may take many cycles to converge, since a shift to one atom will in subsequent cycles require corresponding shifts to neighbours, next-neighbours and so on (Murshudov *et al.*, 1999 ▸).

To enable structures to be refined with weaker or lower resolution data, recent software allow additional restraints to be used relating more distant atoms (Headd *et al.*, 2012 ▸; Nicholls *et al.*, 2014 ▸) or stabilize the refinement through ridge regression (Murshudov *et al.*, 2011 ▸). These approaches may also reduce the rate of convergence.

One common refinement problem arises when solving a structure by molecular replacement, in which a near-homologous structure is placed in the unit cell by rigid-body rotation and translation in order to best explain the observed structure factors. In favourable cases, restrained refinement can resolve the residual differences between the structures; however, for less homologous structures the refinement may not progress (Dodson, 2008 ▸). Domain motions and shifts in chain register are particularly problematic in this regard. Recently, Terwilliger *et al.* (2013 ▸) introduced a technique for ‘model morphing’, in which entire chain segments may be moved based on finding the shift which matches model features within a sphere onto map features, significantly increasing the range of convergence of the refinement in difficult molecular-replacement cases.

The problem of refinement at lower resolutions may also be considered one of parameterization: we are trying to a refine a model described in terms of atomic parameters by fitting a diffraction pattern which cannot possibly resolve atomic features. Some existing approaches address the problem by attaching parameters to structural domains rather than to individual atoms. Rigid-body refinement (Huber & Schneider, 1985 ▸) is often performed within or after molecular replacement, and involves the optimization of the rotation and translation of rigid domains to explain the observed structure factors. Similarly, translation–libration–screw (TLS) refinement seeks to determine the rigid-body motions of a domain which best explain the anisotropic blurring of atomic features in the electron-density map (Driessen *et al.*, 1989 ▸; Winn *et al.*, 2001 ▸). Both methods require that the model be correctly divided into domains in which all of the atoms move, to a first approximation, in a coordinated manner.

In this paper, we outline an alternative approach in which parameters are attached to the grid points of the electron-density map. The shifts to the parameters at any grid point are determined over an extended spherical region around that grid point, the radius of which is chosen so that the shifts will be well determined. The overlap between the spherical regions surrounding neighbouring grid points means that the shifts are highly correlated, and as a result the *effective* number of independent parameters can be low (dependent on the radius of the spherical region), even though the number of grid points is larger than the number of observations (Wang & Shen, 1999 ▸).

### 1.1. Terminology   

The following terminology conventions will be used.

ρ, the current ‘model’ electron-density map.
*D*, the current ‘difference’ density map, based on the disagreement between the observed structure factors and the structure factors calculated from the model map (typically with a weighting term).
*x*, *y*, *z*, fractional coordinates associated with a position in the unit cell. These may be converted to orthogonal coordinates by means of the orthogonalization matrix associated with the unit-cell and axis definitions.
*U*_iso_, isotropic atomic displacement parameter, equivalent to the temperature factor or *B* value scaled by 1/8π^2^.

Other terms will be defined as they are used.

## 2. Theory   

Conventional crystallographic refinement typically involves iterating the following steps.

(i) An electron-density map is calculated from the current atomic model.
(ii) The residual component of the observations that is not explained by the observations is determined.
(iii) The gradient of the residual with respect to the model structure factors leads to difference Fourier coefficients (Murshudov *et al.*, 1997 ▸), or equivalently a difference map (which may be a log-likelihood gradient map in recent implementations).
(iv) The model parameters are adjusted to explain and thus reduce the differences.

The implementation details may vary, and in particular the optimization step may be performed in real or reciprocal space. This paper addresses the final step only, and assumes that an initial electron-density model and the corresponding weighted difference maps have been obtained using existing methods.

In order to parameterize refinement without reference to an underlying atomic model, the parameters may instead be attached to the electron-density map itself. These parameters can be the same as for an atomic model, including positional coordinates and isotropic or anisotropic displacement parameters (*i.e.* temperature factors). While there are no atoms to which to attach these parameters, the electron density is already sampled on a three-dimensional grid; therefore, the parameters will be attached to the same lattice of grid points.

The calculation starts with the current model electron-density map ρ and a difference electron-density map *D* determined from the disagreement between the observed and model structure factors using likelihood weights or other means. These are used to determine a map of shifts, or ‘shift field’, for each parameter to be refined.

Positional parameters may then be refined to make changes to the electron-density map ρ to explain the features of the difference map. For example, if in a region it is found that a shift along the *a* axis will reduce the features of the difference map, the current electron density can be transformed by applying this shift. If different shifts are applied in different parts of the map, the result may lead to a bulk rotation of a region of the electron density.

To determine whether a change in a parameter will explain some features of the difference map *D*, the current electron-density map can be differentiated with respect to that parameter (for example the *x* coordinate). If the resulting gradient map is correlated with the difference map over a particular region, then applying a shift to that coordinate (and therefore to the density) will reduce the features of the difference map. The size of the shift may be determined by finding the shift which best explains the features of the difference map.

Shifts can be determined for each of the (positional and displacement) parameters together to best explain the features of the difference map. However, the difference map also contains noise features, arising in particular from errors in the phases. The noise will lead to errors in the determination of the parameter shifts, which may be reduced by estimating the parameter shifts from larger volumes of the map. The parameter shifts for each grid point in the map will therefore be determined to best explain the features of the difference map in a spherical region around the current grid point, the size of which can be adjusted according to the resolution and the noise in the data. This calculation will be repeated for every grid point in the map.

The parameter shifts are determined by multivariate least-squares regression, and thus scale the shifts to produce a least-squares explanation for the difference-map features. The explanatory variables are the gradients of the model map ρ with respect to each of the parameters, and one additional constant term which will account for any error in the mean of the electron density leading to a constant value in the difference map: this may vary slowly across the unit cell as a result of missing or inaccurate low-resolution reflections.

The least-squares solution for the parameter shifts is given by (1), where **Y** is the vector of difference-map values (from the difference map *D*) for the sphere around the grid point for which the parameter shifts are to be determined, **X** is a matrix whose columns are the gradients of the model map ρ with respect to a given parameter over the corresponding map positions, with one column per parameter, including the constant, and **Δ** is the vector of parameter shifts which best explain the difference map in terms of changes to the current map,
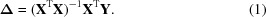
This assumes that all of the map is equally informative in determining the magnitudes of the parameter shifts. However, some regions of the map, in particular the solvent which may not be modelled in the current electron-density map, may be less useful in determining the parameters. This may be addressed by using weighted least-squares regression in which a weight is attached to each observation, given by (2). This weight is expressed as a weight matrix whose diagonal elements correspond to the weights *w* attached to each grid point in the difference map **W** = diag(**w**),
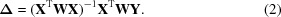
If there are *n* parameters including the constant term, and *m* grid points within the sphere of density, then **X** ^T^ **WX** is an *n* × *n* symmetric matrix whose (*i*, *j*) element is 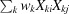. The term **X** ^T^ **WY** is an *n* vector whose *i*th element is 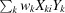.

The vector **Y** is a vector of *m* difference-map values at the *m* grid points within the sphere. In the case where three positional and one isotropic displacement parameter are being refined, the matrix **X** takes the form in (3), where the derivatives are calculated by Fourier transforms (Bricogne, 2001 ▸),
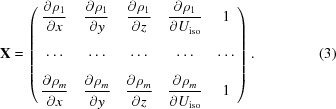
The vector **Δ** contains the shifts to each of the parameters given by (4), where Δ_c_ is the shift to the constant term which is not otherwise used,
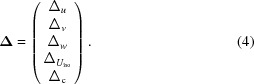
This calculation must be performed for every grid point in the electron-density map ρ. The inversion of a 5 × 5 matrix and matrix-vector product at each grid point are computationally cheap; however, the summation of the products of weight, gradient and difference-map vectors over a spherical region of arbitrary radius about each grid point becomes computationally demanding for larger spheres.

The calculation can be performed in a computationally efficient manner by recognizing that the summation of the various terms over a spherical region around each grid point can be efficiently performed for every grid point in the map simultaneously by convolution. This is similar in concept to the Lifchitz formulation described in Agarwal *et al.* (1981 ▸), except that the gradients are being averaged over a larger sphere rather than over the volume of an individual atom.

If the convolution operator is written as ⊗, then the term 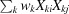 becomes (*w* *_k_* *X_ki_* *X_kj_*)⊗*g*, where *g* is a spherically symmetric function whose radius determines the region over which grid points will contribute to the regression calculation. Similarly, the term 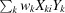 becomes (*w* *_k_* *X_ki_* *Y_k_*)⊗*g*. Convolutions must be performed for each unique element of the *n* × *n* symmetric matrix and the *n* vector. When refining three positional coordinates, one isotropic displacement parameter and the constant, *n* = 5 and therefore 20 convolutions and maps are required. If the convolution is calculated using fast Fourier transforms, the speed of the resulting method is independent of the radius over which the regression is performed.

Once the field of shifts to the *x*, *y*, *z* and *U*_iso_ parameters has been determined, these shifts can be used to update an atomic model by interpolating the value of the shift field for a given parameter using the grid points surrounding the atom. Alternatively, if the model is an electron-density map, the coordinate shifts can be used to interpolate a new map from the existing map (however, the handling of *U*_iso_ is more complex).

## 3. Testing   

Preliminary evaluation of the shift-field approach has been performed for the problem of refining isotropic displacement parameters (*i.e.* temperature factors). Tests were performed using 54 structures for which an atomic model and structure factors were obtained from the Protein Data Bank (Berman *et al.*, 2007 ▸). The test structures were all originally solved by the Joint Center for Structural Genomics (Elsliger *et al.*, 2010 ▸) using a largely automated structure-solution pipeline leading to comparatively uniform data, subject to the different diffraction resolutions of the different crystals.

For each test structure the model was re-refined against the observations using the *REFMAC*5 software (Murshudov *et al.*, 2011 ▸) in order to obtain estimates of the *R* factor and free *R* factor (Brünger, 1993 ▸) using a current version of the software. The isotropic atomic displacement parameters were then set to a constant value (*U*_iso_ = 0.5, *B*_iso_ = 4π^2^), and a zero-cycle run of *REFMAC*5 was used to determine the (higher) *R* factor and free *R* factor for the constant *U*_iso_ model. The results are independent of the chosen constant *U*_iso_ because *REFMAC*5 refines an overall *U*_iso_ even when the model parameters are not modified. The *REFMAC*5 run also produces the model electron-density map ρ and a difference map *D*, which were used in the determination of the shift field.

Shift-field refinement of the model was then performed by iterating the following two steps.

(i) The shift-field calculation was used to determine a map of *U*_iso_ shifts. These shifts were then applied to the atoms of the atomic model by adjusting the model *U*_iso_ by the shift at the corresponding position in the shift map.
(ii) The resulting model was used in a zero-cycle run of *REFMAC*5 to determine new values for the *R* factor and free *R* factor, and to produce updated model and difference maps.

The calculation is iterated for five cycles.

There are three protocol choices to be made in the implementation of the shift-field calculation as presented below.

(i) The radius of the spherical region *r*_0_ [*i.e.* the radius of the function *g*(*r*)] within which the linear regression is performed must be chosen.
(ii) The radial variation of the function *g*(*r*) must be chosen. Three forms were tested: a step function *g*(*r*) = 1, *r *< *r*_0_, a function which declines linearly with distance *g*(*r*) = 1 − *r*/*r*_0_, *r* < *r*_0_, and a function which declines quadratically with distance *g*(*r*) = 1 − (*r*/*r*_0_)^2^, *r* < *r*_0_.
(iii) The gradient and difference-map terms may optionally be masked to exclude unmodelled solvent. The mask *w* excludes grid cells more than 2.5 Å from an atomic centre.

**Figure 1:**
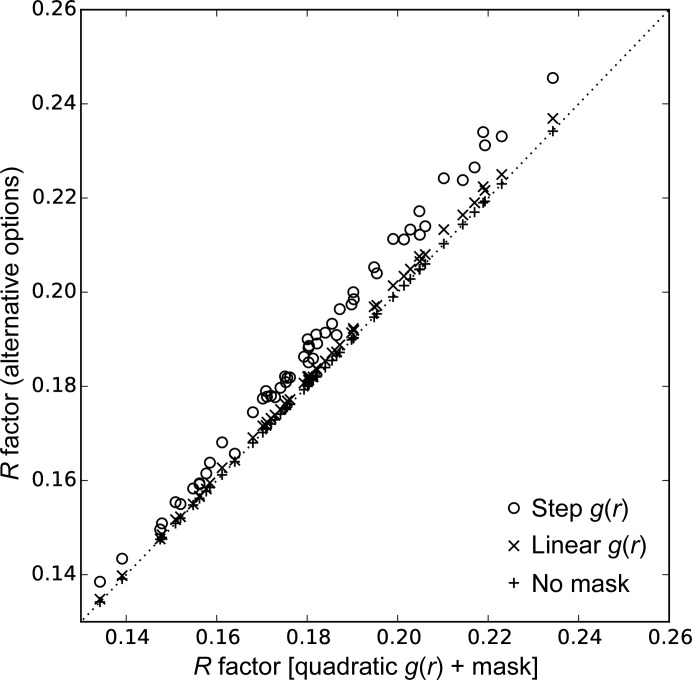
Comparison of the refinement *R* factor with different radial functions and masking. The *x* axis is the *R* factor using the preferred approach of a quadratic form for *g*(*r*) and masking the unmodelled regions. The *y* axis shows the *R* factor using three alternative approaches: ‘o’ for a step function *g*(*r*), ‘×’ for a linear *g*(*r*) and ‘+’ for a quadratic *g*(*r*) but no masking of unmodelled regions. In each case the *R* factor is the best value obtained over trials using ten radii for the radial functions. The quadratic *g*(*r*) outperforms the other forms; however, masking has little effect.

The limiting radius of the radial function *g*(*r*) interacts with the shape of the radial function, so refinement was attempted using multiple combinations of radial function and radius. For each structure in the test set, refinement was attempted with the different radial functions and mask option for ten different values of *r*_0_ between 1.5 and 8 Å, and the best result in terms of *R* factor was retained. The results support the quadratic form for *g*(*r*) (Fig. 1 ▸).

**Figure 2:**
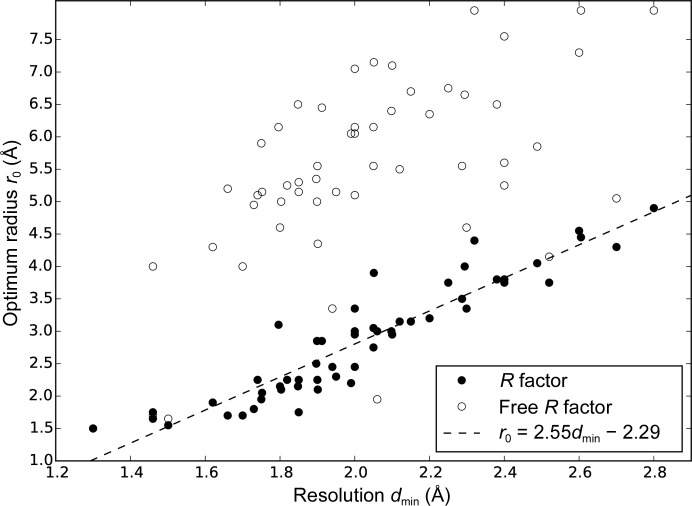
Optimal radius *r*_0_ for the limit of the radial function *g*(*r*) for the test set of 54 structures, plotted against the resolution limit of the observations based on the deposited structure factors. The optimum value of *r*_0_ may be determined on the basis of either the *R* factor or the free *R* factor, as indicated by the closed and open circles.

**Figure 3:**
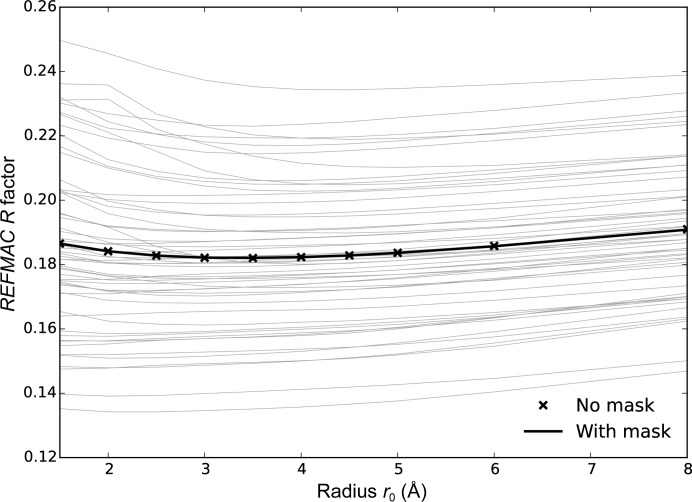
Refinement *R* factor as a function of radius *r*_0_ for the radial function *g*(*r*) for the test set of 54 structures (light lines) using a mask for the unmodelled regions. The mean of the results over the test structures is shown by the solid dark line, while the corresponding mean omitting the mask is shown by the crosses.

The optimal value of *r*_0_ is related to the resolution of the data (Fig. 2 ▸), allowing an appropriate value for *r*_0_ to be selected for an unsolved structure without running multiple calculations with different values of *r*_0_. The best linear fit for *r*_0_ based on the *R* factor is given by (5). Estimation of *r*_0_ to optimize the free *R* factor is preferable and would lead to larger values of *r*_0_, but the relationship is subject to much greater uncertainties.
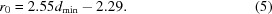
The resulting refinement *R* factor is only very weakly dependent on the radius *r*_0_ of the regression calculation for most of the structures (Fig. 3 ▸). For those structures for which larger radii are optimal, smaller radii do produce significantly worse results; these are generally the structures with the lowest data resolution (*i.e.* the largest *d*_min_; Fig. 2 ▸). The weak increase in *R* factor as *r*_0_ increases past its optimum value suggests that the larger values required at lower resolutions will still lead to useful refinement results.

The masking of unmodelled regions is only expected to make a difference when using larger radii of the regression region, since a small regression region will not significantly overlap the unmodelled region. However, there is no evidence of a benefit from the use of a mask even with larger radii.

**Figure 4:**
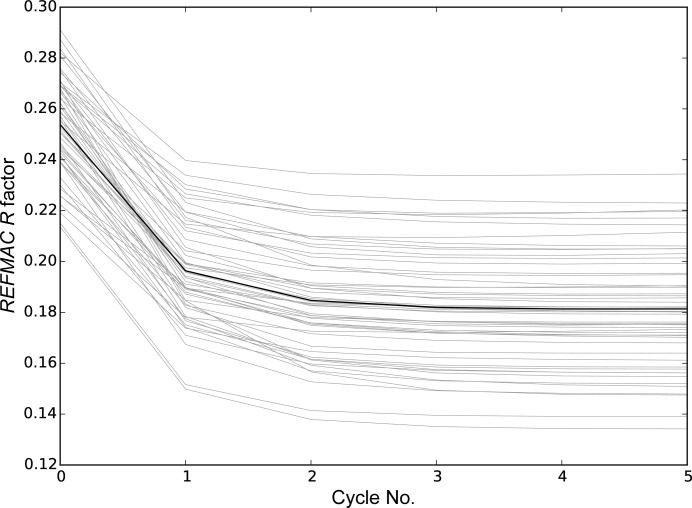
Refinement *R* factor as a function of cycle number for the test set of 54 structures (light lines). The mean of the results over the test structures is shown by the solid dark line.

Most of the improvement in *R* factor is obtained in the first cycle of shift-field refinement; however, some additional improvement is seen over the next two cycles (Fig. 4 ▸). This raises the possibility of using the method as a faster alternative to conventional refinement owing to the fast convergence that is achieved through the omission of chemical restraints.

**Figure 5:**
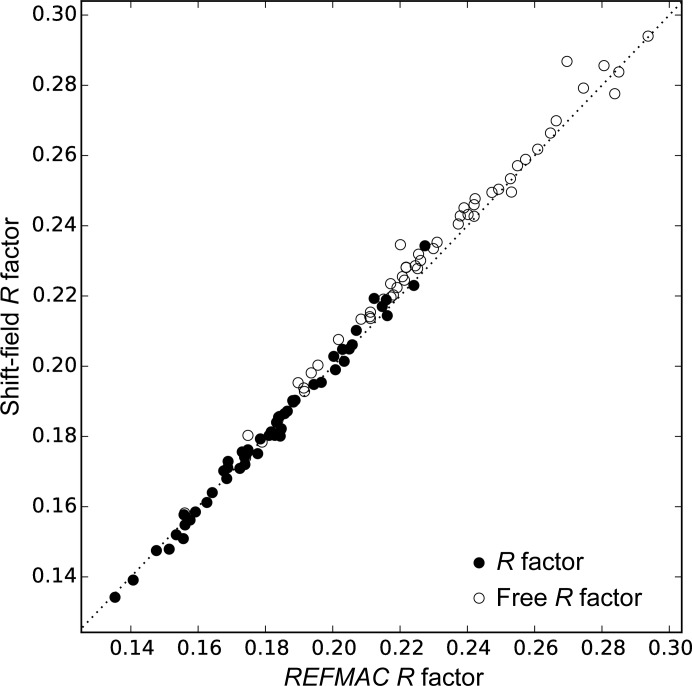
Comparison of the refinement *R* factor when refining from a constant *U*_iso_ model using either the shift-field method or conventional refinement in *REFMAC*5. The *R* factors and free *R* factors from the shift-field method approach the results from *REFMAC*5 without the use of restraints.

The final results of the shift-field refinement are compared with the results of conventional atomic parameter refinement in *REFMAC*5, in each case using the value of *r*_0_ which leads to the best *R* factor (Fig. 5 ▸). The shift-field refinement gives results which are similar to those of conventional refinement, with marginally lower values of the *R* factor and marginally higher values of the free *R* factor. The *R* factors will be artificially lowered by the multiple trials with different values of *r*_0_. If *r*_0_ is selected to optimize the free *R* factor rather than the *R* factor (leading to larger values for *r*_0_), the resulting free *R* factors are on average marginally lower than the values from *REFMAC*5, while the *R* factors are marginally higher.

**Figure 6:**
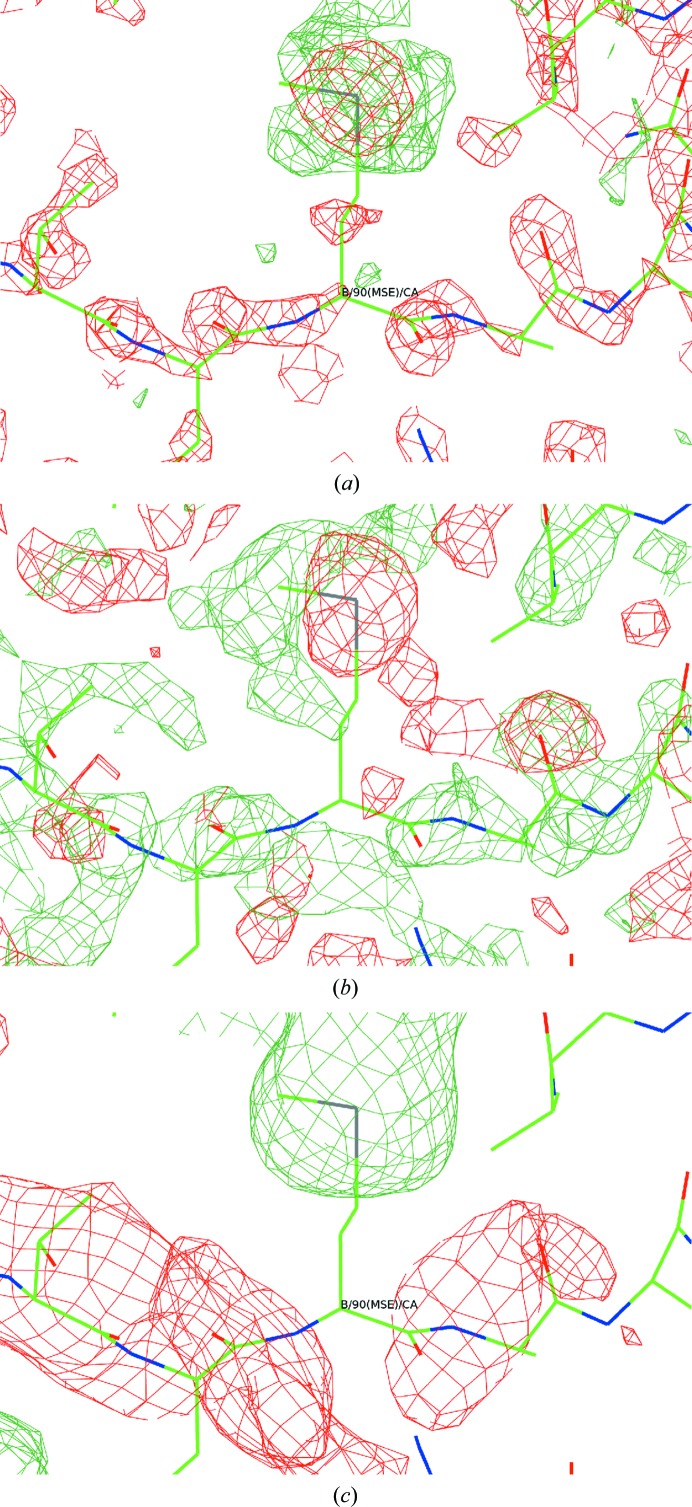
Density-gradient map (*a*), difference map (*b*) and shift field (*c*) for residue B90 of PDB entry 1o6a. The gradient map shows how the density would change for a uniform increase in *B* factor across the map. The difference map shows the changes to the density which would improve agreement with the observations. The shift field shows how the *B* factor would change as a function of position to improve agreement, using *r*_0_ = 3 Å.

The electron-density gradient map, difference map and shift field are shown in Fig. 6 ▸ for the region around a selenomethionine residue in PDB entry 1o6a (Elsliger *et al.*, 2010 ▸). The gradient map shows negative features around atomic centres and positive features in a cage surrounding the Se atom, showing that increasing the *B* factor removes density from the atomic centre and places it around the atom. The difference map is anticorrelated with the gradient map for the main-chain atoms, indicating that their *B* factors should decrease, but is correlated with the gradient map for the Se atom, indicating that its *B* factor should increase. The shift field, which may be crudely understood as the smoothed quotient of these two maps, therefore shows that the *B* factor of the main-chain atoms should decrease and that of the Se atom should increase.

These results provide a sanity check and a preliminary characterization of the behaviour of the method, but do not demonstrate utility for novel problems. In particular, refinement of *U*_iso_ for a model where the coordinates have already been optimized is an easy problem: the shift-field refinement is being performed without restraints, but only one quarter of the number of parameters are being used in comparison to conventional refinement. Realistic evaluation of the utility of the method will only be possible once coordinate refinement has been implemented.

## 4. Discussion   

A new approach to the refinement of an electron-density model against X-ray crystallographic data has been described, which optimizes positional and isotropic displacement parameters on a regular grid, rather than for individual atoms. The parameters are optimized by determining the shifts which will best explain the features of the difference map, and are obtained by multivariate regression over a spherical region around each point on the grid. The radius of the spherical region may be adjusted, and larger radii are required for optimal results at lower resolutions. While the approach has only been tested so far at medium to high resolutions (better than 3 Å), further increasing the radius of the regression region may allow refinement to provide useful results at much lower resolutions.

One potential benefit of shift-field refinement is that it may be used to refine one map against another, even in the absence of an atomic model. This may be useful in the refinement of noncrystallographic symmetry (NCS)-related regions of a map for use in the NCS averaging of flexible domains, or in the fitting of cryo-EM reconstructions to X-ray crystallographic data.

A second potential benefit arises from the calculation of shifts over larger regions. Since groups of atoms move to­gether (because the spherical region around each atom has significant overlap with its neighbours), atoms tend to move in a coordinated fashion without the need for stereochemical restraints. The incorporation of restraints into the refinement calculation produces significant off-diagonal terms in the refinement normal matrix, which reduces the rate of convergence.

Shift-field refinement also has significant limitations which make it complementary to rather than a replacement for conventional refinement. Given that the method produces coordinated shifts to the model parameters over larger regions, it is well suited to the refinement of coordinated changes, for example owing to domain flexibility, but is unsuited to the refinement of noncoordinated changes, such as side-chain orientations or longitudinal shifts in the individual strands of a β-sheet. Conventional refinement, or morphing based on atomic parameters (Terwilliger *et al.*, 2013 ▸), are more appropriate in these cases.

Another limitation arises when the shift field is used to modify a model or map. Variations in the coordinate shift over a region, for example at the boundary of two domains which need to move in different directions, will lead to distortions in the shifted electron density or model. In the model case the refinement may need to be iterated with regularization (Emsley & Cowtan, 2004 ▸) or a fragment-based rebuilding method (Cowtan, 2008 ▸).

The shift-field approach should also be applicable to the refinement of anisotropic displacement parameters. Individual atomic anisotropic displacement parameters can usually only be refined at high resolution since they require six parameters per atom (Murshudov *et al.*, 1999 ▸). At low resolution it is normal to divide the molecule into rigid domains and refine the anisotropic motions which would arise from rigid-body motions of the domains, which are described by a translation–libration–screw (TLS) tensor (Schomaker & Trueblood, 1968 ▸; Winn *et al.*, 2001 ▸). The TLS description has been successful in improving the fit of macromolecular models to observations, but is dependent on the identification of rigid domains and is limited to the description of rigid-body motions or positional uncertainty. Spatially correlated anisotropic displacement parameters can represent the same kind of motion or positional uncertainty as the TLS description (Thorn *et al.*, 2012 ▸). Shift-field refinement may therefore allow the refinement of correlated anisotropic motion for regions of the molecule without assuming rigid-body motion or describing the domains.

### 4.1. Data and methods   

The computer code and data sets used in this paper are available at https://doi.org/10.15124/0dd39199-299c-433e-a29b-1f83a6273fb2.

## Supplementary Material

Methods and data to reproduce the results of the paper. URL: https://doi.org/10.15124/0dd39199-299c-433e-a29b-1f83a6273fb2
